# Preoperative CALLY Index and Postoperative Pathological Axillary Nodal Status in Breast Cancer: Association with Nodal Metastasis and Exploratory Analysis of Observed Pathological Nodal Burden

**DOI:** 10.3390/diagnostics16182934

**Published:** 2026-09-11

**Authors:** Gizem Gunes, Adem Ozcan, Ali Bal, Abdulkadir Unsal

**Affiliations:** Department of Surgical Oncology, Ankara City Hospital, Üniversiteler Mahallesi, Rıfat Börekçi Caddesi No: 1, Çankaya, 06800 Ankara, Türkiye

**Keywords:** breast cancer, CALLY index, clinical nodal assessment, pathological axillary lymph node metastasis, observed pathological nodal burden, systemic inflammation

## Abstract

**Background/Objectives:** The C-reactive protein–albumin–lymphocyte (CALLY) index integrates systemic inflammation, nutritional status, and host immunity. We evaluated its association with postoperative pathological axillary nodal status and its incremental discrimination beyond routinely recorded preoperative clinical factors. **Methods:** This retrospective single-center diagnostic-accuracy study screened 380 patients evaluated between July 2019 and January 2026. The primary cohort included 247 unilateral M0 patients undergoing upfront surgery; 80 neoadjuvant-treated M0 patients were analyzed separately. CALLY was calculated from laboratory values obtained within 7 days before surgery. Associations and discrimination were assessed using logistic regression, Firth penalized logistic regression when clinical nodal status caused separation, paired bootstrap AUC comparisons, restricted cubic splines, and prespecified sensitivity analyses for BMI and available inflammatory/comorbidity covariates. **Results:** In the primary cohort, 75 patients (30.4%) were pN-positive. Median CALLY was 2.629 in pN-positive and 3.586 in pN0 patients (*p* = 0.058), and standalone discrimination was weak (AUC = 0.576; 95% CI, 0.496–0.651). In the age- and cT-adjusted model, the OR per CALLY doubling was 0.89 (95% CI, 0.72–1.10; *p* = 0.271). Preoperative cN status nearly separated the outcome; in a Firth model including age, cT, cN, and CALLY, the CALLY OR was 0.78 (profile 95% CI, 0.51–1.21; *p* = 0.250). Adding CALLY to age, cT, and cN changed the AUC from 0.988 to 0.988 (ΔAUC = −0.0001; 95% CI, −0.0013 to 0.0041; *p* = 0.551). No nonlinearity was detected (*p* = 0.352), and BMI/confounder sensitivities were concordant. Secondary neoadjuvant and observed-burden analyses remained exploratory and imprecise. **Conclusions:** In this single-center upfront-surgery cohort, preoperative CALLY was not independently associated with postoperative axillary nodal positivity and provided no convincing incremental discrimination beyond the available clinical assessment. The data do not support using CALLY alone for axillary staging or surgical decisions; limited high-burden event counts preclude a definitive exclusion of smaller associations.

## 1. Introduction

Breast cancer is a biologically heterogeneous disease in which postoperative pathological axillary lymph node status remains central to anatomical staging, prognostic assessment, and selection of locoregional and systemic treatment [1,2]. Although contemporary axillary management has shifted toward less extensive surgery in selected patients, the distinction between node-negative disease, limited nodal involvement, and high nodal burden continues to influence regional treatment decisions.

Physical examination, axillary ultrasonography, magnetic resonance imaging, and positron emission tomography/computed tomography contribute to preoperative axillary assessment, but no single modality provides perfect discrimination [2]. This has encouraged investigation of inexpensive blood-based biomarkers that may complement conventional assessment. Such markers are attractive when they can be obtained from routine preoperative laboratory tests without additional cost or specialized infrastructure.

Cancer-related inflammation can facilitate angiogenesis, invasion, immune escape, and metastatic dissemination [3]. C-reactive protein (CRP) reflects systemic inflammatory activity, serum albumin is influenced by inflammation and nutritional status, and circulating lymphocytes contribute to antitumor immune surveillance [4]. The CRP–albumin–lymphocyte (CALLY) index integrates these three domains. A lower value indicates a less favorable inflammatory, nutritional, and immune profile. The index was initially developed and validated as a prognostic biomarker after hepatectomy for hepatocellular carcinoma [5]. It has subsequently been evaluated in other solid tumors, including colorectal and gastric cancer [4,6].

Although the prognostic relevance of CALLY may be explained by its reflection of the host’s systemic inflammatory, nutritional, and immune status, its potential relationship with current disease extent requires a separate rationale. Greater tumor burden and metastatic dissemination may be accompanied by increased tumor-associated inflammation, impaired lymphocyte-mediated antitumor immunity, and inflammation-related metabolic alterations. Therefore, a composite index incorporating CRP, albumin, and lymphocyte count could theoretically be associated with pathological nodal involvement. However, CALLY should not be regarded as a direct staging test or as a replacement for axillary imaging, image-guided needle biopsy, or sentinel lymph node biopsy. Its potential value would be limited to serving as an inexpensive adjunctive risk marker if it provided meaningful discrimination or incremental information beyond established preoperative clinical factors. The present study was designed to test this hypothesis rather than to assume clinical utility.

In breast cancer, available evidence has mainly focused on prognosis, stage, and treatment response. A low preoperative CALLY index has been associated with worse disease-free and overall survival after surgery [7]. Subsequent studies have examined its prognostic role in stage III disease, its association with pathological complete response after neoadjuvant treatment, and its relationship with overall disease stage [8,9,10]. These endpoints differ from prediction of postoperative axillary nodal metastasis. A small diagnostic-accuracy study of 59 patients reported essentially no discrimination for axillary nodal metastasis and no association with metastatic lymph node count [11].

The present study therefore evaluated the association and discriminatory performance of the preoperative CALLY index for postoperative pathological axillary lymph node metastasis and observed pathological nodal burden. To preserve a one-patient, one-tumor, one-axilla analytic unit and reduce treatment-related heterogeneity, unilateral patients with M0 disease undergoing upfront surgery were designated as the primary cohort. Patients receiving neoadjuvant systemic therapy were analyzed separately according to postoperative ypN status. In response to the clinically relevant question of incremental value, CALLY was also evaluated beyond the available preoperative clinical axillary assessment.

## 2. Materials and Methods

### 2.1. Study Design and Patient Population

This retrospective, single-center observational study screened patients with histologically confirmed breast cancer who entered the preoperative surgical evaluation pathway at the Surgical Oncology Clinic of Ankara City Hospital between July 2019 and January 2026. Patients who proceeded to surgery and met the laboratory eligibility criteria formed the surgical source cohort. Data were obtained from the hospital information system, electronic medical records, laboratory database, preoperative anesthesia assessments, operative reports, and final postoperative histopathology reports.

This study was reported in accordance with the Standards for Reporting Diagnostic Accuracy Studies (STARD) 2015 recommendations, where applicable [12].

Patient selection was performed sequentially using clinically and methodologically predefined criteria that were applied independently of CALLY values and postoperative nodal outcomes. A total of 380 patients with primary invasive epithelial breast carcinoma entered the institutional preoperative surgical evaluation pathway during the study period and were identified through clinical, pathology, and preoperative assessment records. Operative and final pathology records were subsequently reviewed for patients who proceeded to surgery. Patients with pure ductal carcinoma in situ, recurrent breast cancer, or non-epithelial breast malignancies were not eligible. Laboratory eligibility was then assessed within this clinically identified preoperative surgical population. Five patients were excluded because CRP was reported below the assay’s lower limit of quantification without an exact numerical value, preventing reliable calculation of the CALLY index. Eleven patients were excluded because elevated CRP at the relevant preoperative assessment was accompanied by suspected active infection or acute inflammation. Eight patients were excluded because elevated CRP led to deferred anesthetic clearance; these patients did not return to our center for repeat assessment or subsequent surgery, and therefore no later clinically valid preoperative laboratory measurements were available. After these 24 exclusions, the source cohort comprised 356 patients with complete preoperative albumin, absolute lymphocyte count, and an exact quantitative CRP result suitable for CALLY calculation.

After source-cohort formation, 15 patients with bilateral breast cancer were excluded to preserve a one-patient, one-tumor, one-axilla analytical unit. Five additional patients were excluded because postoperative axillary pathology was not evaluable: two were classified as ypNX, and three had no examined axillary lymph nodes despite a pN0 database entry.

This left 336 unilateral patients with evaluable postoperative axillary pathology. Nine patients with distant metastatic disease at diagnosis were excluded from the M0 analyses because systemic metastatic disease could independently influence inflammatory and nutritional parameters. The final M0 analytic population therefore consisted of 327 patients.

**Figure 1:**
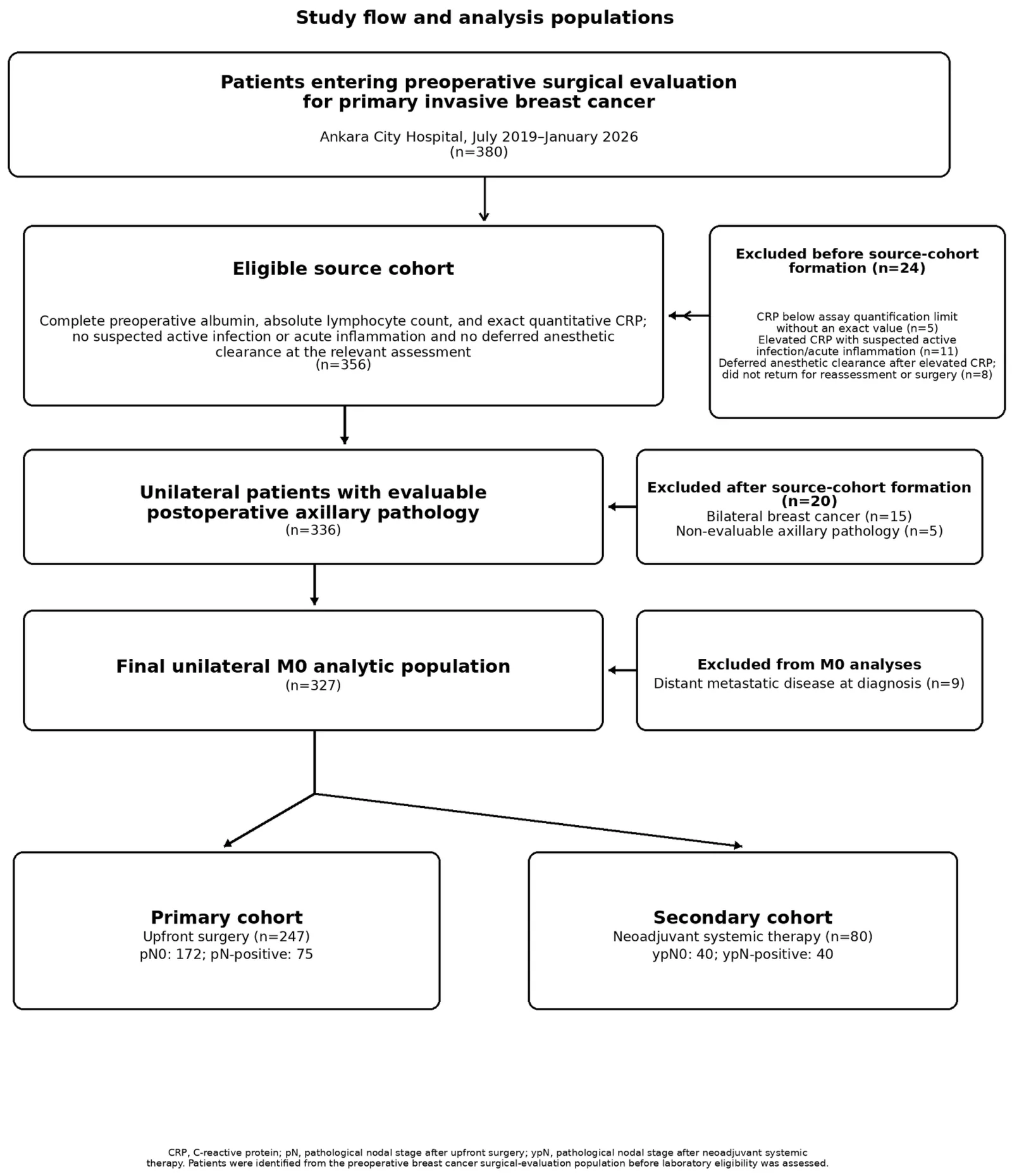
Study flow and analysis populations.

The primary cohort comprised 247 unilateral patients with M0 disease who underwent upfront surgery. A separate secondary cohort comprised 80 unilateral patients with M0 disease who received neoadjuvant systemic therapy before surgery. Neoadjuvant-treated patients were analyzed separately rather than excluded to avoid combining untreated pN status with post-treatment ypN status. No formal a priori sample-size calculation was performed; all eligible patients within the study period were included. The study flow is presented in Figure 1.

### 2.2. Clinical and Pathological Variables

Collected variables included age, sex, body mass index (BMI), clinical T category, preoperative clinical/radiological axillary status, distant metastasis at diagnosis, neoadjuvant treatment, type of breast and axillary surgery, postoperative tumor size, number of excised and positive lymph nodes, histological type, histological grade, estrogen receptor (ER), progesterone receptor (PR), human epidermal growth factor receptor 2 (HER2), Ki-67 proliferation index, lymphovascular invasion, perineural invasion, multifocality, and multicentricity. The axillary variable recorded at diagnosis was confirmed by the authors to derive from preoperative physical examination and/or ultrasonography and was analyzed as cN0 versus cN-positive. Detailed ultrasound morphology and image-guided axillary-biopsy results were not separately abstracted. Clinical and pathological staging was based on the eighth edition of the American Joint Committee on Cancer (AJCC) TNM staging system. Postoperative pathological variables, including histological type and receptor findings, were obtained from the final surgical pathology report.

Preoperative BMI was calculated as weight in kilograms divided by height in meters squared using height and weight recorded in the clinical or anesthesia assessment closest to surgery. BMI was analyzed as a continuous variable in regression. Categories of <25.0, 25.0–29.9, and ≥30.0 kg/m^2^ were used only for descriptive and exploratory comparisons of CALLY distributions.

Histological type and immunohistochemical molecular phenotype were treated as distinct pathological characteristics. Histological type referred to categories such as invasive carcinoma of no special type/ductal carcinoma, invasive lobular carcinoma, and other special histological types. Histological type was derived from the final surgical pathology specimen and was not entered into the preoperative prediction models.

Molecular phenotype was derived from ER, PR, and HER2 results recorded in the final surgical resection pathology report. Although receptor testing was routinely performed on diagnostic core-biopsy specimens as part of care, the pretreatment results were not separately abstracted in the research dataset and could not be reconstructed reliably. The available surgical-specimen receptor values were therefore used only for postoperative description and were excluded from preoperative models to avoid information leakage. Tumors expressing ER and/or PR without HER2 overexpression or amplification were classified as HR-positive/HER2-negative; HER2-positive and triple-negative phenotypes were defined using standard criteria.

This design prevents postoperative receptor information from being treated as a preoperative predictor, but the absence of separately abstracted pretreatment core-biopsy receptor status limits the clinical completeness of the prediction models.

### 2.3. Preoperative Laboratory Measurements and CALLY Calculation

For patients who proceeded to surgery, the most recent serum albumin, CRP, and absolute lymphocyte count measurements obtained within 7 days before surgery were retrieved from the institutional laboratory system. CRP was measured by a turbidimetric method (institutional reference interval, 0–5 mg/L). Suspected active infection or acute inflammation was identified from contemporaneous clinical documentation at the relevant preoperative assessment rather than by a CRP-only threshold. Five patients had a report below the assay’s lower limit of quantification without an exact concentration; the exact numerical lower limit and the individual excluded values were not retained in the analysis files. Because CRP is the denominator of CALLY, assigning zero is undefined and substituting an arbitrary fraction of an unknown limit can create extreme CALLY values. These five patients were therefore excluded without imputation. Eleven patients with documented suspected infection/acute inflammation and eight whose anesthetic clearance was deferred after elevated CRP were also outside the 356-patient source dataset; their detailed covariates and outcomes were unavailable for post hoc descriptive comparison. This selection mechanism is addressed as a limitation.

Albumin was recorded in g/dL, CRP in mg/L, and absolute lymphocyte count in ×10^9^/L. CALLY was recalculated uniformly as albumin (g/dL) × absolute lymphocyte count (×10^9^/L)/CRP (mg/L). In neoadjuvant-treated patients, these measurements represented the post-treatment assessment within 7 days before surgery and were analyzed only in the separate secondary cohort.

### 2.4. Postoperative Pathological Nodal Outcomes

The primary outcome was postoperative pathological axillary nodal positivity. Postoperative pathological nodal status was determined from the final histopathological examination of sentinel lymph node biopsy and/or axillary lymph node dissection specimens. Patients with a recorded positive-node count of at least one were classified as pathologically node-positive, whereas patients with a recorded positive-node count of zero and at least one examined axillary lymph node were classified as node-negative. Because isolated tumor cell status was not separately available in the retrospective database, nodal classification was based on the recorded positive-node count in the final pathology report.

The CALLY index was calculated retrospectively and was not available to, or used by, the treating team when determining the type or extent of axillary surgery. Axillary management was based on routine clinical, imaging, pathological, and intraoperative assessment. The pathologists who assessed the axillary specimens were unaware of the retrospectively calculated CALLY values.

The secondary outcome was the observed postoperative pathological nodal burden among node-positive patients, categorized as low burden (1–3 positive lymph nodes) or high burden (≥4 positive lymph nodes). Axillary surgical management consisted of sentinel lymph node biopsy and/or axillary lymph node dissection. Because the extent of axillary surgery may influence the number of metastatic lymph nodes identified, nodal-burden analyses were considered exploratory. In patients who underwent sentinel lymph node biopsy without completion axillary lymph node dissection, the recorded positive-node count represented the observed pathological nodal burden rather than the total anatomical axillary burden. Because high-burden events were infrequent, burden analyses were performed separately in the upfront-surgery and neoadjuvant cohorts.

### 2.5. Statistical Analysis

Continuous variables were summarized as mean ± standard deviation or median (interquartile range), and categorical variables as n (%). Welch’s *t* test, Mann–Whitney U test, χ^2^ test, Fisher’s exact test, or the Fisher–Freeman–Halton exact test was used as appropriate. Table 1 comparisons were descriptive and their *p* values exploratory; no multiplicity adjustment was applied.

CALLY was right-skewed and was log_2_-transformed for regression; its odds ratio therefore represents a two-fold increase. Standalone discrimination for nodal positivity was summarized by the AUC with 2000 bootstrap resamples. The prespecified conventional logistic model included age per 10 years, clinical T category, and log_2_ CALLY. Postoperative variables were excluded from preoperative prediction models.

A clinical comparator model added the preoperative cN0/cN-positive variable to age and cT. Because all pN-positive events occurred among cN-positive patients, conventional maximum-likelihood logistic regression exhibited quasi-complete separation. Firth bias-reduced penalized logistic regression was therefore used, with profile penalized-likelihood confidence intervals and *p* values for CALLY. The cN coefficient itself was not interpreted as a stable effect estimate under this separation pattern.

Incremental discrimination was evaluated by comparing age+cT and age+cT+CALLY as a secondary comparator and, more importantly, age+cT+cN versus age+cT+cN+CALLY. Paired AUC differences, 95% confidence intervals, and two-sided *p* values were obtained from 3000 outcome-stratified bootstrap resamples. Brier scores were also calculated for the cN-based models.

Potential nonlinearity was examined with a restricted cubic spline for CALLY using four knots at the 5th, 35th, 65th, and 95th percentiles. The nonlinear spline terms were tested jointly with a likelihood-ratio test. Because the spline was exploratory and the extremes were sparse, the fitted curve was interpreted together with its confidence interval.

BMI was entered continuously in a sensitivity model. Additional residual-confounding analyses used the available preoperative variables: smoking status, BMI, a cardiometabolic count (diabetes, hypertension, and coronary artery disease), and an inflammation modifier indicating corticosteroid exposure in the laboratory window, antibiotic exposure, autoimmune disease, or hematologic disease. A restricted-cohort sensitivity excluded patients with any inflammation modifier. Liver disease and nonsteroidal anti-inflammatory drug use were not available as analysis variables.

The secondary neoadjuvant cohort was analyzed separately for residual ypN positivity because its CALLY measurement was post-treatment. Treatment regimens were summarized descriptively. Analyses of observed pathological nodal burden (1–3 versus ≥4 positive nodes) were treatment-stratified, univariable, and explicitly hypothesis-generating because of the small number of high-burden events.

Axillary procedures were categorized as SLNB only or ALND-containing; modified radical mastectomy was classified as ALND-containing. An observed-burden sensitivity analysis was restricted to node-positive patients with an ALND-containing procedure.

After the analytic cohorts were derived, no additional patients were excluded for missing model covariates or outcomes. BMI was complete in the primary cohort. No imputation was performed. Analyses were performed using Python, version 3.11 (Python Software Foundation, Beaverton, OR, USA), with the open-source SciPy, statsmodels, Patsy, and scikit-learn packages; all tests were two-sided. ChatGPT (GPT-6 Astra OpenAI, San Francisco, CA, USA; accessed in July–August 2026) assisted with statistical code development. All code and numerical outputs were reviewed and verified by the authors.

### 2.6. Ethical Approval

The study protocol was approved by the Ankara City Hospital Clinical Research Ethics Committee No. 1 on 6 May 2026 (approval number: TABED-26-2538). The study was conducted in accordance with the Declaration of Helsinki. The requirement for individual informed consent was waived by the ethics committee because of the retrospective design and the use of anonymized data.

## 3. Results

### 3.1. Cohort Formation and Primary-Cohort Characteristics

A total of 380 patients with primary invasive breast cancer entered the preoperative surgical-evaluation pathway. Five patients with nonquantifiable CRP, 11 with documented suspected infection/acute inflammation, and eight with deferred anesthetic clearance were excluded before formation of the 356-patient quantitative-laboratory source cohort. Patient-level covariates for these 24 pre-source exclusions were not present in the analysis files. After exclusion of 15 bilateral cases and five patients without evaluable axillary pathology, 336 unilateral patients remained; nine M1 patients were excluded from the M0 analyses. The final M0 analytic population comprised 327 patients: 247 treated with upfront surgery and 80 treated with neoadjuvant systemic therapy followed by surgery.

In the primary upfront-surgery cohort, 172 patients (69.6%) were pN0 and 75 (30.4%) were pN-positive. Preoperative assessment classified 164 (66.4%) as cN0 and 83 (33.6%) as cN-positive. All 75 pN-positive patients were cN-positive; among pN0 patients, 164 were cN0 and eight were cN-positive (Fisher’s exact *p* < 0.001). Among cN0 patients, 143 underwent SLNB only and 21 an ALND-containing procedure; among cN-positive patients, 30 underwent SLNB only and 53 an ALND-containing procedure. Detailed ultrasound morphology and biopsy status were unavailable, so cN-positive denotes the recorded suspicious clinical/radiological assessment rather than necessarily biopsy-proven disease. The mean age was 58.7 ± 11.4 years and mean BMI 24.18 ± 2.11 kg/m^2^. Detailed characteristics are shown in Table 1.

### 3.2. Preoperative CALLY and Postoperative Pathological Nodal Positivity

**Figure 2:**
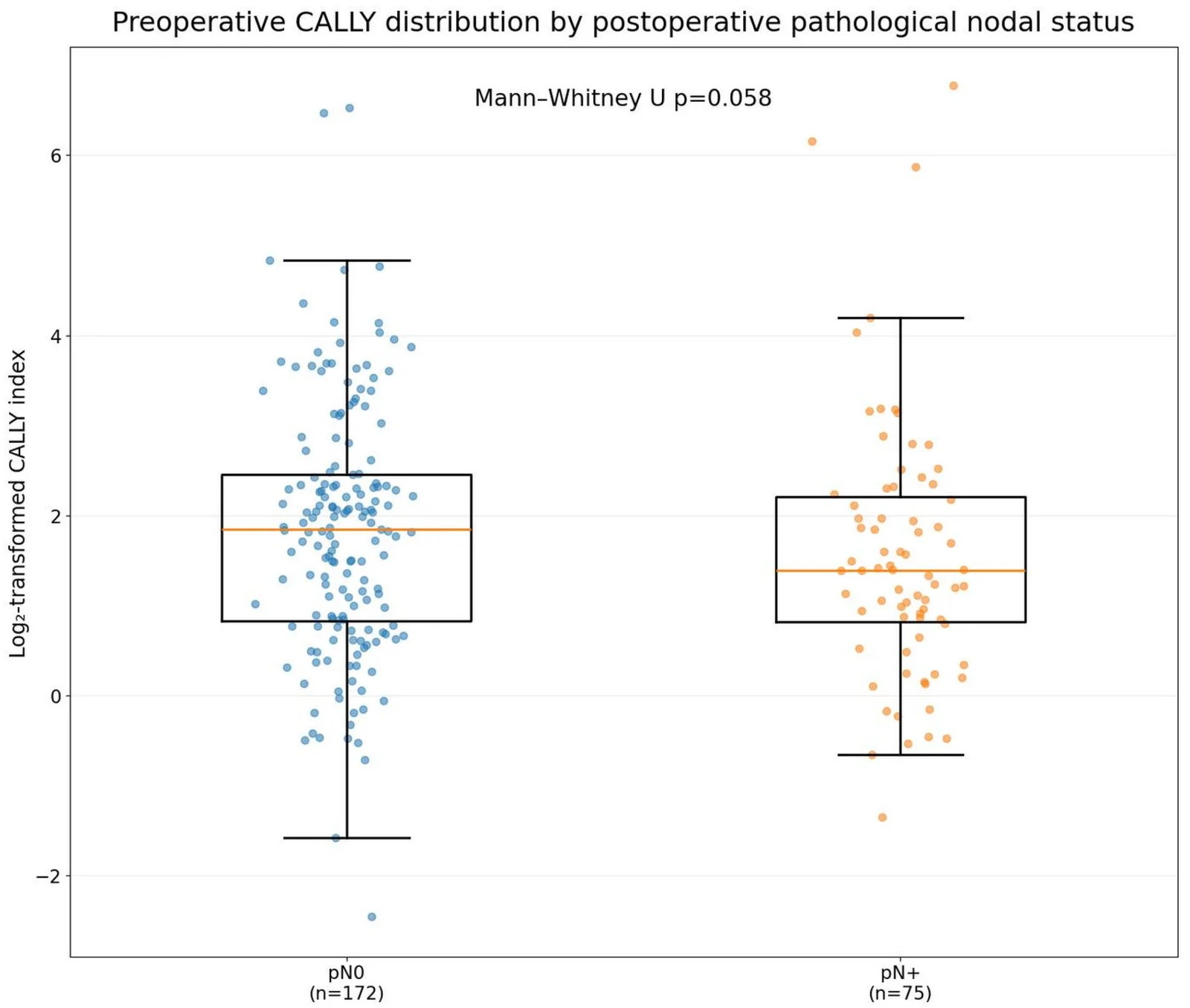
Log_2_-transformed preoperative CALLY distribution by postoperative pathological nodal status.

Median preoperative CALLY was 3.586 (IQR, 1.773–5.479) in pN0 patients and 2.629 (IQR, 1.770–4.624) in pN-positive patients. Although CALLY tended to be lower in the node-positive group, the difference did not reach statistical significance (Mann–Whitney *p* = 0.058) (Figure 2). Median lymphocyte count was also lower in pN-positive patients (1.81 vs. 1.94 ×10^9^/L, *p* = 0.053), and CRP was higher (3.50 vs. 2.50 mg/L, *p* = 0.059), whereas albumin was similar between groups (*p* = 0.695). In the complementary parametric analysis, mean log_2_-transformed CALLY was 1.83 ± 1.39 in pN0 patients and 1.56 ± 1.46 in pN-positive patients. The mean difference was 0.27 (95% CI, −0.12 to 0.67), and Welch’s *t* test was not statistically significant (*p* = 0.174).

Each two-fold increase in CALLY was not associated with postoperative nodal positivity in univariable analysis (OR, 0.87; 95% CI, 0.71–1.06; *p* = 0.165) or in the prespecified model adjusted for age and cT (OR, 0.89; 95% CI, 0.72–1.10; *p* = 0.271). In the Firth model including age, cT, preoperative cN status, and CALLY, the CALLY OR was 0.78 per doubling (profile 95% CI, 0.51–1.21; *p* = 0.250) (Table 2). Restricted cubic splines provided no evidence of nonlinearity (*p* = 0.352; Figure S1). Surgical-specimen ER/PR/HER2 values were not entered because they were postoperative, and pretreatment core-biopsy receptor data were unavailable.

In the fully adjusted sensitivity model containing age, cT, BMI, smoking, cardiometabolic count, and the inflammation modifier, the CALLY OR was 0.865 (95% CI, 0.695–1.078; *p* = 0.197). After excluding 23 patients with an inflammation modifier, the corresponding OR was 0.914 (95% CI, 0.728–1.149; *p* = 0.443) (Table S2).

### 3.3. Discrimination and Incremental Value

**Figure 3:**
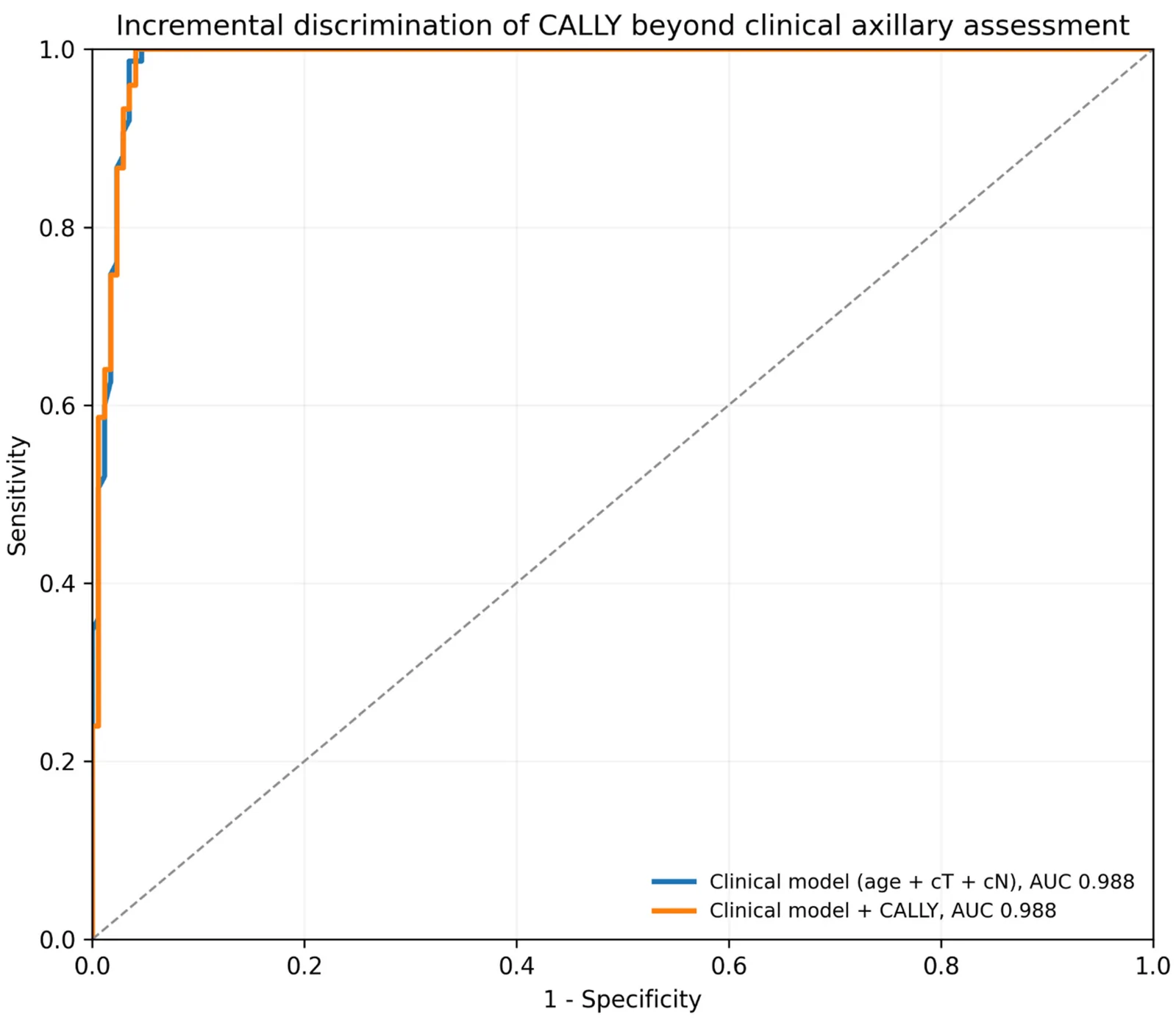
Incremental discrimination of CALLY beyond clinical axillary assessment. Receiver operating characteristic curves for the clinical model (age, clinical T category, and preoperative cN status) and the clinical model plus CALLY in the primary cohort. The curves nearly overlap; the paired ΔAUC was −0.0001 (95% CI, −0.0013 to 0.0041; *p* = 0.551).

CALLY alone showed weak discrimination (AUC, 0.576; 95% CI, 0.496–0.651). The age+cT model had an AUC of 0.670, which increased to 0.698 after adding CALLY (ΔAUC, 0.027; 95% CI, −0.007 to 0.069; *p* = 0.280); this secondary comparison provided no convincing evidence of clinically meaningful incremental discrimination. In the clinically more complete comparison, cN status produced near-separation: the age+cT+cN model had an AUC of 0.9883 (95% bootstrap CI, 0.9793–0.9984) and the model plus CALLY had an AUC of 0.9882 (95% CI, 0.9802–0.9990). The paired ΔAUC was −0.0001 (95% CI, −0.0013 to 0.0041; *p* = 0.551), with Brier scores of 0.0271 and 0.0260, respectively (Figure 3; Table S2). Thus, the available clinical axillary assessment dominated discrimination and CALLY did not add measurable ranking performance.

### 3.4. Neoadjuvant Cohort

The secondary neoadjuvant M0 cohort comprised 80 patients, of whom 40 (50.0%) had residual ypN-positive disease. Regimens included TCHP/other anti-HER2-based therapy in 41 patients, AC→T in 26, and AC→T with or without carboplatin in 13; nine received immunotherapy. Median post-treatment preoperative CALLY was 2.408 in ypN0 and 1.525 in ypN-positive patients (*p* = 0.156), with an AUC of 0.593 (95% CI, 0.467–0.723) and OR per doubling of 0.86 (95% CI, 0.68–1.09; *p* = 0.205) (Table 3). These results describe a heterogeneous post-treatment biomarker and were not compared directly with untreated-patient performance.

### 3.5. Exploratory Observed Pathological Nodal-Burden Analyses

Observed high pathological nodal burden was uncommon. In the upfront-surgery cohort, seven of 75 node-positive patients had ≥4 recorded positive nodes; the AUC was 0.433 (95% CI, 0.214–0.685) and the OR per CALLY doubling was 1.19 (95% CI, 0.74–1.92; *p* = 0.472).

In the neoadjuvant cohort, 11 of 40 ypN-positive patients had ≥4 recorded positive nodes; the AUC was 0.476 (95% CI, 0.265–0.690) and the OR per doubling was 1.14 (95% CI, 0.80–1.64; *p* = 0.462) (Table 4). These hypothesis-generating analyses found no evidence of discrimination, but their wide intervals cannot exclude clinically relevant associations.

### 3.6. Axillary Procedure and ALND-Restricted Sensitivity Analyses

In the primary cohort, 173 patients underwent SLNB without ALND and 74 underwent an ALND-containing procedure. Median CALLY was 3.542 (IQR, 1.800–5.594) in the SLNB-only group and 2.636 (IQR, 1.717–4.493) in the ALND-containing group (Mann–Whitney *p* = 0.138). Comparison of log_2_-transformed CALLY values using Welch’s *t* test was also non-significant (*p* = 0.586). After adjustment for clinical T category, CALLY was not associated with ALND-containing surgery (adjusted OR per two-fold increase, 0.97; 95% CI, 0.79–1.20; *p* = 0.801). In contrast, higher clinical T category was associated with ALND-containing surgery (cT2 vs. cT1: adjusted OR, 3.57; 95% CI, 1.92–6.65; *p* < 0.001; cT3–4 vs. cT1: adjusted OR, 11.30; 95% CI, 3.82–33.46; *p* < 0.001) (Table S1).

Among 45 node-positive patients who underwent an ALND-containing procedure, 38 had 1–3 positive nodes and seven had at least 4 positive nodes. Median CALLY was 2.636 and 2.964, respectively (*p* = 0.702). The AUC for observed high pathological nodal burden was 0.451 (95% bootstrap CI, 0.222–0.705), and the OR per two-fold increase in CALLY was 1.08 (95% CI, 0.66–1.79; *p* = 0.754). Thus, restricting the analysis to patients with more extensive axillary sampling did not reveal an association between CALLY and observed high pathological nodal burden (Table S1).

## 4. Discussion

In this primary cohort of unilateral M0 patients undergoing upfront surgery, CALLY showed weak standalone discrimination and was not independently associated with pN positivity. CALLY was evaluated against the available preoperative axillary assessment. cN status nearly separated pN outcomes, and adding CALLY to age, cT, and cN changed the AUC by essentially zero. The profile-likelihood interval around the Firth CALLY estimate included both a potentially protective association and no association, so the result is best interpreted as no convincing evidence of incremental value in this cohort rather than proof of absence.

The biological rationale for CALLY remains plausible because CRP, lymphocytes, and albumin reflect inflammation, immunity, and nutritional reserve. Population-based research has also associated CALLY with mortality among patients with cancer [13]. Nevertheless, a nonspecific systemic index need not track regional anatomical dissemination. Prior breast-cancer studies mainly evaluated prognosis or response [7,8,9,10]. However, the more directly comparable small axillary study also reported negligible discrimination [11]. Related prognostic research has evaluated the axillary lymph node ratio in nonmetastatic triple-negative breast cancer and plasma-only circulating tumor DNA assays for minimal residual disease detection and recurrence prediction [14,15]. These studies address prognosis and recurrence, which differ from the preoperative nodal-staging question examined here. Our data therefore support separating the prognostic hypothesis from the narrower question of preoperative nodal staging.

The cN-based result changes the clinical interpretation. Sixty-six percent of the primary cohort was cN0 and none of these patients was pN-positive in the recorded dataset, whereas 75 of 83 cN-positive patients were pN-positive. This unusually strong relationship explains the AUC near 0.99 and the separation that required Firth regression. It also shows that CALLY did not improve the available clinical assessment. However, cN was recorded only as a binary physical-examination/ultrasound variable; detailed morphology, standardized reporting, and axillary-biopsy results were unavailable. Previous studies have examined abnormal-node counts on ultrasound as predictors of axillary burden and radiomics derived from ultrasound, MRI, and mammography for predicting axillary nodal metastasis [16,17,18]. The study therefore cannot address incremental value beyond a fully specified contemporary imaging-and-biopsy pathway.

BMI is relevant to the interpretation of circulating CRP concentrations [19]. BMI and the available confounder analyses were concordant with the main result. Neither adjustment for BMI nor additional control for smoking, cardiometabolic comorbidity, corticosteroid/antibiotic exposure, autoimmune disease, and hematologic disease materially changed the CALLY estimate. Excluding patients with recorded inflammation modifiers also yielded a null result. These analyses reduce, but do not eliminate, concern about residual confounding because liver disease, nonsteroidal anti-inflammatory drug use, nutritional-screening scores, weight loss, and body-composition measures were unavailable.

The neoadjuvant analysis should not be interpreted as a baseline biomarker study. CALLY was measured after systemic treatment and shortly before surgery, and the cohort included cytotoxic chemotherapy, anti-HER2 therapy, carboplatin, corticosteroid exposure inherent to some regimens, and immunotherapy. Treatment can affect every CALLY component and residual nodal response. Research on pembrolizumab and event-free survival in early triple-negative breast cancer addresses treatment outcomes [20]. The present exploratory analysis assessed post-treatment CALLY in relation to residual nodal status. The neoadjuvant findings are therefore secondary, exploratory, and not directly comparable with the untreated cohort.

The observed pathological nodal-burden analyses were also limited by the small number of high-burden events. Only seven upfront-surgery and 11 neoadjuvant patients had ≥4 recorded positive nodes, producing wide confidence intervals. In addition, SLNB-only sampling does not measure total anatomical axillary burden. The absence of a signal is hypothesis-generating and cannot rule out a clinically relevant association; even the ALND-restricted sensitivity remained imprecise.

The study’s strengths include its one-patient/one-axilla primary cohort, separation of treatment settings, continuous modeling of CALLY, explicit incremental-discrimination analysis, avoidance of a data-derived cut-off, and multiple sensitivity analyses. The adjusted CALLY interval in the age+cT model (0.72–1.10 per doubling) makes a large adverse association unlikely in this sample, but smaller effects remain compatible with the data. For the observed-burden endpoint, precision was insufficient for a comparable exclusion.

First, selection and spectrum bias remain possible. The 24 exclusions preceding the 356-patient source cohort were not represented in the analysis files, so their characteristics could not be compared post hoc. Excluding nonquantifiable CRP and clinically invalid inflammatory assessments may have removed extremes of the CALLY distribution. The results apply to surgical patients with exact quantitative CRP and clinically accepted preoperative laboratory values.

Second, residual confounding is possible despite the sensitivity analyses. Available smoking, cardiometabolic, medication-window, autoimmune, and hematologic variables were used, but other inflammatory, hepatic, nutritional, and medication factors were not uniformly recorded.

Third, the preoperative comparator was incomplete. The binary cN variable was available, but detailed ultrasound morphology and biopsy confirmation were not. Pretreatment core-biopsy ER, PR, and HER2 status was also not separately abstracted. The surgical-specimen receptor values could not be substituted without introducing postoperative information into a preoperative model.

Fourth, measurement limitations include retrospective laboratory timing, unavailable exact CRP lower-limit information for excluded patients, and possible unrecognized subclinical inflammation. Restricted cubic splines did not identify nonlinearity, although estimates at the CALLY extremes were imprecise.

Fifth, the observed-burden and neoadjuvant analyses had limited event counts and no external validation. Future multicenter work should prospectively standardize cN imaging/biopsy variables, retain pretreatment receptor data, define below-quantification handling in advance, document inflammatory modifiers, and evaluate calibration and decision benefit in addition to AUC.

## 5. Conclusions

In this single-center cohort of unilateral M0 patients undergoing upfront breast cancer surgery, preoperative CALLY showed weak standalone discrimination and was not independently associated with postoperative pathological axillary nodal positivity. Adding CALLY provided no convincing incremental discrimination beyond the available age, cT, and binary preoperative cN assessment. Secondary neoadjuvant and observed-burden analyses were exploratory and insufficiently precise to exclude smaller or context-specific associations. CALLY should not be used alone to guide axillary staging or surgery in this setting.

## Figures and Tables

**Table 1:** Clinicopathological characteristics of the primary unilateral M0 upfront-surgery cohort according to postoperative pathological nodal status.

Variable	pN0 (n = 172)	pN-Positive (n = 75)	*p* Value
Age, years	58.7 ± 11.2	58.7 ± 11.7	0.958
BMI, kg/m^2^	24.23 ± 2.13	24.05 ± 2.07	0.536
**Sex**			0.587
Female	170 (98.8)	73 (97.3)	
Male	2 (1.2)	2 (2.7)	
**Clinical T category**			<0.001
cT1	103 (59.9)	23 (30.7)	
cT2	63 (36.6)	39 (52.0)	
cT3–4	6 (3.5)	13 (17.3)	
**Preoperative clinical/radiological axilla**			<0.001
cN0	164 (95.3)	0 (0.0)	
cN-positive	8 (4.7)	75 (100.0)	
**Histological type (surgical specimen)**			0.577
Invasive carcinoma of no special type/ductal carcinoma	144 (83.7)	61 (81.3)	
Invasive lobular carcinoma	25 (14.5)	11 (14.7)	
Other special/mixed histological types	3 (1.7)	3 (4.0)	
**Molecular phenotype (surgical specimen)**			0.003
HR-positive/HER2-negative	143 (83.1)	51 (68.0)	
HER2-positive	22 (12.8)	12 (16.0)	
Triple-negative	7 (4.1)	12 (16.0)	
**Histological grade**			0.020
Grade 1	37 (21.5)	10 (13.3)	
Grade 2	106 (61.6)	41 (54.7)	
Grade 3	29 (16.9)	24 (32.0)	
**Lymphovascular invasion**			<0.001
No	143 (83.1)	34 (45.3)	
Yes	29 (16.9)	41 (54.7)	
**Perineural invasion**			0.160
No	164 (95.3)	68 (90.7)	
Yes	8 (4.7)	7 (9.3)	
**Multifocality**			0.347
No	158 (91.9)	66 (88.0)	
Yes	14 (8.1)	9 (12.0)	
Postoperative tumor size, mm	18.0 (13.8–28.3)	28.0 (18.0–40.0)	<0.001
Albumin, g/dL	4.48 (4.20–4.67)	4.50 (4.20–4.63)	0.695
Lymphocyte count, ×10^9^/L	1.94 (1.69–2.37)	1.81 (1.51–2.20)	0.053
CRP, mg/L	2.50 (1.50–4.40)	3.50 (1.80–4.60)	0.059
CALLY index	3.586 (1.773–5.479)	2.629 (1.770–4.624)	0.058

Data are presented as mean ± standard deviation, median (interquartile range), or n (%). All *p* values in this descriptive table are exploratory and were not adjusted for multiple comparisons. Preoperative cN status was derived from physical examination and/or ultrasonography. Histological type and molecular phenotype were derived from the final surgical specimen and were not included as preoperative predictors. BMI, body mass index; CRP, C-reactive protein; CALLY, C-reactive protein–albumin–lymphocyte index; HER2, human epidermal growth factor receptor 2; HR, hormone receptor.

**Table 2:** Logistic regression analyses for postoperative pathological nodal positivity in the primary cohort, including the clinical comparator.

Variable	Unadj. OR	95% CI	*p* Value	Adjusted OR	95% CI	*p* Value
Age per 10 years	1.01	0.79–1.28	0.957	1.02	0.79–1.31	0.866
cT2 vs. cT1	2.77	1.52–5.07	0.001	2.84	1.54–5.21	<0.001
cT3–4 vs. cT1	9.70	3.34–28.22	<0.001	9.13	3.12–26.72	<0.001
CALLY index per two-fold increase	0.87	0.71–1.06	0.165	0.89	0.72–1.10	0.271
CALLY per doubling, Firth clinical model	—	—	—	0.78	0.51–1.21	0.250

Adjusted estimates in the upper panel are from conventional logistic regression including age, cT, and CALLY. The Firth clinical model additionally included preoperative cN status; its CALLY confidence interval and *p* value are profile penalized-likelihood results. In all 247 primary-cohort patients, median BMI was 24.0 kg/m^2^ (IQR, 23.0–25.0; range, 20.0–32.0). BMI was not associated with nodal positivity (OR per kg/m^2^, 0.971; 95% CI, 0.842–1.119; *p* = 0.682), and BMI adjustment did not materially alter CALLY (OR, 0.886; 95% CI, 0.717–1.095; *p* = 0.263; Table S1).

**Table 3:** Treatment-stratified analyses of preoperative CALLY for postoperative pathological nodal positivity.

Cohort	n/Events	CALLY Node-Negative	CALLY Node-Positive	*p* Value	AUC (95% CI)	OR per Doubling (95% CI), *p*
Primary: unilateral M0, upfront surgery	247/75	3.586 (1.773–5.479)	2.629 (1.770–4.624)	0.058	0.576 (0.496–0.651)	0.87 (0.71–1.06), *p* = 0.165
Secondary: unilateral M0, neoadjuvant therapy	80/40	2.408 (1.071–5.188)	1.525 (0.715–3.782)	0.156	0.593 (0.467–0.723)	0.86 (0.68–1.09), *p* = 0.205

CALLY values are median (interquartile range). In the neoadjuvant cohort, node-positive denotes postoperative residual ypN-positive disease. AUC, area under the receiver operating characteristic curve; CI, confidence interval; OR, odds ratio. In the neoadjuvant cohort, CALLY measurements represented the post-neoadjuvant, preoperative assessment.

**Table 4:** Exploratory treatment-stratified analyses of preoperative CALLY and observed postoperative pathological nodal burden among node-positive patients.

Cohort	n (1–3/≥4)	CALLY, 1–3 Nodes	CALLY, ≥4 Nodes	*p* Value	AUC (95% CI)	OR per Doubling (95% CI), *p* Value
Upfront surgery	75 (68/7)	2.621 (1.696–4.772)	2.964 (2.437–4.084)	0.574	0.433 (0.214–0.685)	1.19 (0.74–1.92), 0.472
Neoadjuvant therapy	40 (29/11)	1.547 (0.740–3.761)	1.503 (0.698–3.756)	0.832	0.476 (0.265–0.690)	1.14 (0.80–1.64), 0.462

CALLY values are median (interquartile range). Observed high pathological nodal burden was defined as at least 4 positive lymph nodes. These analyses were exploratory because of the limited event counts. AUC, area under the receiver operating characteristic curve; CI, confidence interval; OR, odds ratio. AUCs were calculated with lower CALLY values specified as indicating a higher probability of the outcome; values below 0.50 indicate discrimination in the direction opposite to the prespecified hypothesis.

## Data Availability

The data presented in this study are available on request from the corresponding author due to institutional and ethical restrictions.

## Supplementary Materials

The following supporting information can be downloaded at: https://www.mdpi.com/article/10.3390/diagnostics16182934/s1, Table S1: BMI-adjusted, axillary procedure, and ALND-restricted sensitivity analyses; Table S2: Clinical-comparator, nonlinearity, and residual-confounding analyses; Figure S1: restricted cubic spline assessment of CALLY.
