# Supplementary material for: Preoperative CALLY Index and Postoperative Pathological Axillary Nodal Status in Breast Cancer: Association with Nodal Metastasis and Exploratory Analysis of Observed Pathological Nodal Burden
Source: Diagnostics (Basel). 2026 Sep 11;16(18):2934. doi: 10.3390/diagnostics16182934 (PMC13605056; doi:10.3390/diagnostics16182934)
Supplement: Supplementary file 1 [file diagnostics-16-02934-s001.zip › diagnostics-4510806-supplementary.pdf]

Supplementary Materials

Preoperative CALLY Index and Postoperative Pathological Axillary Nodal Status in Breast Cancer: Association with Nodal Metastasis and Exploratory Analysis of Observed Pathological Nodal Burden

Gizem Gunes, Adem Ozcan, Ali Bal, and Abdulkadir Unsal

Table S1. BMI-adjusted, axillary-procedure, and ALND-restricted sensitivity analyses.

Analysis	Estimate / comparison	95% CI / secondary detail	p value
**A. BMI-adjusted primary model**			
BMI, per 1 kg/m² increase	Adjusted OR 0.971	0.842–1.119	0.682
CALLY, per two-fold increase	Adjusted OR 0.886	0.717–1.095	0.263
Likelihood-ratio test for adding BMI	$\chi^2 = 0.170$	—	0.680
**B. Axillary-procedure analysis**			
CALLY: SLNB only vs ALND-containing	3.542 (1.800–5.594) vs 2.636 (1.717–4.493)	Mann–Whitney U	0.138
$\log_2$(CALLY): SLNB only vs ALND-containing	1.778 ± 1.385 vs 1.667 ± 1.486	Welch t test	0.586
CALLY, per doubling	Adjusted OR 0.97	0.79–1.20	0.801
cT2 vs cT1	Adjusted OR 3.57	1.92–6.65	<0.001
cT3–4 vs cT1	Adjusted OR 11.30	3.82–33.46	<0.001
**C. ALND-restricted observed-burden sensitivity**			
CALLY: 1–3 vs ≥4 positive nodes	2.636 (1.931–4.670) vs 2.964 (2.437–4.084)	Mann–Whitney U	0.702
AUC for ≥4 positive nodes	0.451	0.222–0.705	—
CALLY, per doubling	OR 1.08	0.66–1.79	0.754

Data are presented as median (interquartile range), mean ± standard deviation, adjusted odds ratios (ORs), or area under the curve (AUC), as applicable. The axillary-procedure analysis included 173 patients treated with SLNB only and 74 with an ALND-containing procedure. The ALND-restricted observed-burden analysis included 45 node-positive patients: 38 with 1–3 and 7 with ≥4 positive nodes. CI, confidence interval; SLNB, sentinel lymph node biopsy; ALND, axillary lymph node dissection.

Table S2. Clinical-comparator, nonlinearity, and residual-confounding analyses.

Analysis	Estimate	95% CI / detail	p value
**A. Clinical axillary distribution**			
cN0	164 pN0 / 0 pN+	Overall cN-by-pN association	<0.001*
cN-positive	8 pN0 / 75 pN+	—	—
**B. Firth age+cT+cN+CALLY model**			
CALLY, per doubling	OR 0.782	0.512–1.205	0.250
**C. Incremental discrimination**			
Clinical model (age+cT+cN)	AUC 0.9883	0.9793–0.9984	—
Clinical model + CALLY	AUC 0.9882	0.9802–0.9990	—
Paired ΔAUC	−0.0001	−0.0013 to 0.0041	0.551
Brier score: clinical model	0.0271	—	—
Brier score: clinical model + CALLY	0.0260	—	—
**D. Restricted cubic spline**			
Joint test of nonlinear terms	$\chi^2 = 2.09$ (2 df)	Knots: 0.765, 2.199, 4.318, 16.361	0.352
**E. Residual-confounding sensitivity analyses**			

Analysis	Estimate	95% CI / detail	p value
Fully adjusted sensitivity model: CALLY per doubling	OR 0.865	0.695–1.078	0.197
Restricted cohort (n = 224): CALLY per doubling	OR 0.914	0.728–1.149	0.443

*Fisher's exact test for the overall association between binary preoperative cN status and postoperative pN status. The clinical model contained age, clinical T category, and binary preoperative cN status; the extended model added log_2-transformed CALLY. Because cN produced quasi-complete separation, the CALLY OR, CI, and p value were estimated using Firth bias-reduced logistic regression with profile penalized-likelihood inference. AUC confidence intervals and ΔAUC inference were based on 3,000 outcome-stratified bootstrap resamples. The restricted cubic spline used four knots at CALLY values of 0.765, 2.199, 4.318, and 16.361. The fully adjusted sensitivity model included age, cT, BMI, smoking, cardiometabolic count, and an inflammation modifier. The restricted cohort excluded patients with any corticosteroid or antibiotic exposure during the laboratory window or with an autoimmune or hematologic disease flag.

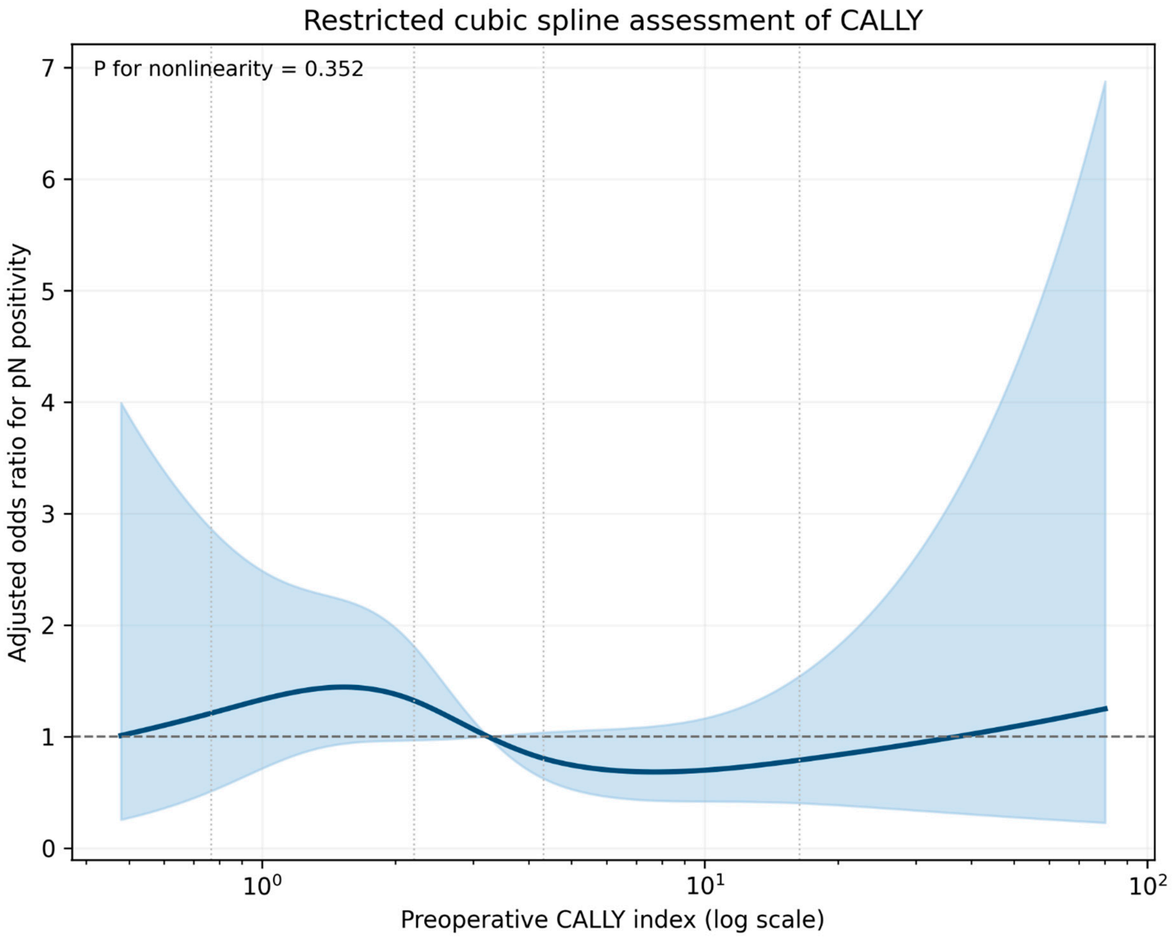

Figure S1. Restricted cubic spline assessment of the age- and cT-adjusted association between preoperative CALLY and pN positivity in the primary cohort. The curve is referenced to CALLY = 0.5; shaded bands are 95% confidence intervals. Vertical dotted lines show the prespecified knot locations. The test for nonlinearity was not significant (p = 0.352), and estimates were imprecise at the distributional extremes.
